# Psychometric properties of dementia attitudes scale, dementia knowledge assessment tool 2 and confidence in dementia scale in a Greek sample

**DOI:** 10.1002/nop2.546

**Published:** 2020-07-03

**Authors:** Mara Gkioka, Magdalini Tsolaki, Sotirios Papagianopoulos, Birgit Teichmann, Despina Moraitou

**Affiliations:** ^1^ Network Aging Research University of Heidelberg Heidelberg Germany; ^2^ School of Medicine Aristotle University of Thessaloniki Thessaloniki Greece; ^3^ 1st Department of Νeurology AHEPA University Hospital Thessaloniki Greece; ^4^ 3rd Department of Neurology Papanikolaou General Hospital Thessaloniki Greece; ^5^ Laboratory of Psychology Section of Experimental & Cognitive Psychology School of Psychology Aristotle University of Thessaloniki Thessaloniki Greece

**Keywords:** assessment, attitudes, dementia care, education programme, knowledge, nurse education, psychometrics, self‐efficacy, student, validity

## Abstract

**Aim:**

Τo validate the Greek version of the Dementia Knowledge Assessment Tool 2, the Dementia Attitudes Scale and Confidence in Dementia Scale.

**Design:**

A quantitative cross‐sectional design was applied for translation and validation. The STROBE checklist for observational research has been followed to this survey.

**Method:**

Two hundred and twelve students from the School of Psychology (Aristotle University of Thessaloniki). Psychometric properties were assessed through construct validity (principal component analysis), internal consistency (Cronbach's alpha) and convergent validity.

**Results:**

High internal reliability was found for Confidence in Dementia Scale (α = 0.85), adequate reliability for Dementia Attitudes Scale (α = 0.74) and acceptable reliability for Dementia Knowledge Assessment Tool 2 (α = 0.68). Construct validity was satisfactory for Dementia Attitudes Scale (two factors: social comfort and knowledge). The convergent validity was supported to this survey. All three tools are reliable and valid to measure knowledge, confidence and attitudes towards dementia in Greek research context.

## 1 INTRODUCTION

Dementia is as a major public health problem due to the longer life expectancy and the lack of efficient therapeutic strategies (Prince et al., 2013). People with dementia (PwD) are more likely to be admitted to general hospitals than people of similar age without dementia, and they are vulnerable to poor outcomes related to admissions (Prince, Comas‐Herrera, Knapp, Guerchet, & Karagiannidou, 2016). Professional caregivers have often little knowledge about dementia and a lack of confidence in PwD care (Elvish et al., 2014). Poor knowledge among healthcare professionals may lead to a delayed diagnosis (Knopman, Donohue, & Gutterman, 2000) and to misrepresentation of symptoms, resulting to inappropriate treatment (Edwards, Plant, Novak, Beall, & Baumhover, 1992).

Due to a significant increase in number of patients suffering from dementia, The World Alzheimer Report 2016 provides important recommendations to strengthen healthcare systems (Prince et al., 2016). The global dementia plan aims to improve the awareness and friendliness of the disease. Thus, dementia trainings for clinical staff in hospital settings are steadily increasing (Dewing & Dijk, 2014). Greece enacted the national dementia strategy in 2014 including the education of professional caregivers in hospital settings (action 2, axis 7 for the Greek national plan).

Staff involved in dementia care vary greatly regarding their formal training and their knowledge on the disease (Spector, Orrell, Schepers, & Shanahan, 2012). Successful training programmes are usually evaluated for their overall impact, which is a critical issue in the field of human resource development (HRD). According to Holton's model, an intervention has to be evaluated in three levels regarding the outcomes: learning, individual performance and organization (Holton III, 1996). Self‐efficacy has been shown to be related to training outcomes (Holton III, 1996). Specifically, beliefs about self‐efficacy determine how much effort will be expended and whether coping behaviour in challenging situations will be initiated (Bandura, 1977), thus suggesting that confidence is likely to be an important factor in staff behaviour (Elvish et al., 2014). Level of knowledge is argued to be linked to confidence (Hughes, Bagley, Reilly, Burns, & Challis, 2008). Thus, it is also important to measure knowledge (Elvish et al., 2014). Successful learners are expected to feel confident and therefore more motivated to transfer the acquired knowledge (Holton III, 1996). The trainee's attitude towards the organization and the job also has an impact on their motivation. An attitude is a response to a person, object or event that combines three components: emotional, cognitive and behavioural and each of these carries a pleasurable to displeasing effect, a favourable to unfavourable cognition and a supportive to hostile behaviour (Breckler, 1984). It is likely that employees with more positive job attitudes are more motivated to learn, demonstrating positive training outcomes. Thus, knowledge, confidence and positive attitudes are correlated with each other and are important measurements for a training outcome at workplace (e.g. a healthcare facility).

## 2 BACKGROUND

### 2.1 Existing measures

There are numerous self‐report tools for measuring knowledge, attitudes and confidence or self‐efficacy in provision of caring PwD, but most of them have been developed by researchers for the purpose of particular studies (Teodorczuk, Mukaetova‐Ladinska, Corbett, & Welfare, 2014) (Hobday, Gaugler, & Mittelman, 2017) (Galvin et al., 2010) (Palmer et al., 2014). Such scales are useful but lack validation in different samples and the evidence of convergent and divergent validity. Using a standardized tool for evaluating training outcomes will help avoid biased results and conclusions, increasing the methodological strength of studies (Burgio et al., 2001). Thus, in healthcare facilities, it is important to use measurement scales of good quality, with established psychometric properties (Spector et al., 2012).

Regarding knowledge, there are many validated tools. In particular, the Alzheimer's Disease Knowledge Test (ADKT) (Dieckmann, Zarit, Zarit, & Gatz, 1988) is widely used due to its adequate reliability and validity and it is capable of assessing knowledge about Alzheimer's disease among laypeople, patients, caregivers and professionals. Alzheimer's Disease Knowledge Scale (ADKS) is an updated version of ADKT. Although ADKS has a limited use (Scerri, 2017) (Kimzey, Mastel‐Smith, & Alfred, 2016), it exhibits adequate to good psychometric properties. Besides, a recent confirmatory factor analysis suggests that it may be used to effectively generate overall knowledge scores, when administered to nurses, family members of PwD, health workers, students in health‐related disciplines and general adult population (Annear et al., 2017). Moreover, ADKS has already been validated in the Greek population with a marginal internal consistency (Prokopiadou et al., 2013). Dementia Quiz (DQ) is another tool developed in UK (Gilleard & Groom, 1994) and appears most suitable for family caregivers, achieving adequate reliability and validity with this group. Broad use of this measure requires significant modifications (Spector et al., 2012). The University of Alabama Alzheimer's Disease Knowledge Test (UAB‐ADKT) (Barrett, Haley, Harrell, & Powers, 1997) tool was constructed to test knowledge in healthcare professionals. However, further studies are needed for evidence in construct validity. Moreover, it has a limited number of items, focusing on biomedical domains and thus it is unlikely to be suitable for populations beyond medical staff and nurses. The KAML‐C (Kuhn, King, & Fulton, 2005) tool demonstrates good content and face validity. However, its specific focus on the early stages of AD may be limiting. Moreover, further validity testing is required because of the small sample size and the lack of variation in the sample. The recent Knowledge in Dementia (KIDE) Scale (Elvish et al., 2014) has been used to evaluate staff training and has been validated.

There are many scales for attitudes in older people, but only few specifically for people with dementia. Lintern and colleagues developed the Approaches to Dementia Questionnaire (ADQ) (Lintern & Woods, 2000) which measures hopefulness and person‐centred approaches. The ADQ has since been used with nurse staff in general hospitals (Surr, Smith, Crossland, & Robins, 2016) (Smythe et al., 2014) (Banks et al., 2013). Regarding self‐efficacy scales, the Inventory of Geriatric Nurse Self‐Efficacy (Mackenzie & Peragine, 2003) had good psychometric properties used in staff of care homes. The Self‐Perceived Behavioural Management Self‐Efficacy Profile (Schindel Martin & Dupuis, 2005) (SBMSEP) is another scale tested for its validity and reliability and measures the confidence in accomplishing tasks in the clinic, as well as the behaviour of caregivers and their ability to manage the emotional stress experienced by PwD. Moreover, the Caring Efficacy Scale (Coates, 1997) (CES) assesses staff beliefs about their own caring efficacy in terms of their ability to express a caring attitude and develop caring relationships with their patients. It has been reported a high internal consistency and good construct validity.

Greece lacks validated questionnaires for use in healthcare settings, even though dementia is a major health problem. In the current study, we intended to test the Dementia Knowledge Assessment Tool (DKAT2), the Confidence in Dementia (CODE) Scale and the Dementia Attitudes Scale (DAS) because all three demonstrated good psychometric properties in their original version and are more suitable for use by professional caregivers. Although a knowledge tool (ADKS) has already validated in Greek, we chose DKAT2 because it evaluates the overall knowledge of dementia and dementia care, rather than any specific dementia‐related disease (e.g. Alzheimer's disease) bridging the gap between existing measures (Toye et al., 2013). The DKAT2 can also help to establish realistic requirements and outcomes of education programmes (Toye et al., 2013). Moreover, although the Approaches in Dementia Questionnaire (ADQ) is commonly used (Smythe et al., 2014) (Banks et al., 2013) (Kang, Moyle, & Venturato, 2011), research community lacks a more general scale constructed via construct mapping (O’Connor & McFadden, 2010). Thus, we chose the DAS questionnaire for current validation because it reflects the three components of attitudes (affection, behaviour and cognition) towards individuals with AD and related dementias. Additionally, there are many self‐efficacy tools, as already mentioned, but there is a lack of tools related to confidence in care. CODE was developed to measure confidence in working with people with dementia and it has been already stressed its good psychometric properties (Elvish et al., 2014). Its prospects of implementation as an evaluation tool of a dementia training programme in general hospitals make us to choose this for validation in Greek population.

The aim of this cross‐sectional survey is to examine the psychometric properties of these three questionnaires (DKAT2, DAS and CODE) about knowledge, confidence and attitudes towards dementia in Greek population, to be used as evaluation tools in a dementia training programme for nursing and other staff which will be conducted in Greek general hospitals.

#### 2.1.1 Research question

Are these three tools reliable and valid to the Greek context according to their internal consistency, construct validity and convergence validity?

## 3 DESIGN

A quantitative cross‐sectional study design was applied to validate the three translated scales. The present survey adheres to EQUATOR guidelines of reporting research using the “Strengthening the Reporting of Observational Studies in Epidemiology” (STROBE) checklist (File S1).

## 4 METHOD

### 4.1 Participants

In the current study, adult students of the School of Psychology (Aristotle University of Thessaloniki) took part, who enrolled in the cross‐sectional study about the adaptation of the DKAT2, CODE Scale and DAS. Due to practical restrains, a convenience sample of students was selected, which is a commonly used technique (Ho et al., 2020). Although students are not a representative sample of the general population because they are young and highly educated, they are easy to contact and reach, available and willing to participate. Thus, participants were recruited by a first‐year course of cognitive psychology, where one of the authors (D.M.) was the tutor. One author (M.G.) visited the students during the class (240 students in total) and announced the purpose of the study. Participants were eligible if they were aged 18+ years, if their mother language was Greek and if they were enrolled into a full‐time undergraduate degree programme. All participants were recruited in one day (May 2019). Two hundred twenty (220) students expressed the willingness to participate voluntarily, and they signed a written consent. Two hundred twenty (212) students returned back the questionnaires. Young students came from many areas of Greece, and they were predominantly female (90%). Ages ranged from 18–41 (*M* = 23.5, *SD* = 5.7). We followed the methodological recommendations regarding the sample size needed for validation studies. Thus, to calculate sample size, ten people per item were required (Polit, 2008). Since the DKAT2 had the greatest length among the used questionnaires (21 items), a sample size of 210 was required (21*10). Thus, our 212 participants considered as adequate sample.

### 4.2 Procedure

The course instructor (D.M.) offered students the chance to complete the scales before class started. Students completed the 21 items of DKAT2, the 9 items of CODE and the 20 items of DAS. In DAS, participants additionally responded to the question: “Have you ever known or cared for someone with Alzheimer Disease or Related Disorders (ADRD)?” This question was included to examine whether familiarity with ADRD correlated with positive attitudes. The whole procedure took 15–20 min for students to complete them.

### 4.3 Measures

#### 4.3.1 Dementia Knowledge Assessment Tool (DKAT2)—a 21‐item questionnaire

The “Dementia Knowledge Assessment Tool 2” was developed to provide a reliable, valid and feasible tool to measure care workers’ foundation level knowledge of dementia (Toye et al., 2013). The DKAΤ2 addressed two conceptual domains: “dementia and its progress” and “support and care.” According to its developers, DKAT2, addressed in family and staff carers, is a unidimensional questionnaire. Its internal consistency reliability (Cronbach's alpha coefficient) is promising (0.79). Items included statements like: “Blood vessel disease can also cause dementia” and “Only older adults develop dementia.” Response options were yes/no/don't know. Thirteen items were correct statements and 8 items were incorrect. There is a reversal rating, and the final score is derived from the sum of all correct items. Higher score indicates higher knowledge in dementia topic. This tool can provide an indication of overall knowledge, and it is feasible for use in aged care environments to establish training needs and necessary information resources. Moreover, it helps to evaluate knowledge of all dementia‐related illness, making the tool more broadly applicable (Toye et al., 2013).

#### 4.3.2 Confidence in Dementia (CODE) Scale—a 9‐item questionnaire

The “Confidence in Dementia Scale” was developed to measure hospital staff confidence in working with PwD (Elvish et al., 2014). According to its developers, CODE is an unidimensional questionnaire and its internal consistency reliability (Cronbach's alpha coefficient) is very good (0.91). Items included statements like: “I feel able to manage situations when a person with dementia becomes agitated.” Response options were scored on a 5‐point Likert scale with anchored ratings of “not confident,” “somewhat confident” and “very confident.” This meant that the total score ranged between 9–45, with a higher score representing better confidence in working with PwD. Cut‐off points in the scale are as follows: 0–18 not confident, 19–35 somewhat confident and 36–45 very confident (Elvish et al., 2018). The overall Kaiser–Meyer–Olkin (ΚΜΟ) was 0.90, suggesting that the scale has good internal consistency without too much item redundancy and a good sample adequacy. Thus, DAS psychometric properties support its use as a research tool.

#### 4.3.3 Dementia Attitudes Scale (DAS)—a 20‐item questionnaire

The Dementia Attitudes Scale (DAS) was developed to measure attitudes towards dementia for both college students and direct care workers (family and professional carers) (O’Connor & McFadden, 2010). According to its developers, DAS essentially has a two‐factor structure and its total internal consistency reliability (Cronbach's alpha coefficient) ranged from 0.83–0.85. The first factor (α = 0.82) labelled as “dementia knowledge” consisting from statements like “People with ADRD can be creative.” The second factor (α = 0.75) labelled as “social comfort” including statements like “I feel confident around people with ADRD.” These two factors were correlated, *r* = 0.29, *p <* .01, and together explained 38.72% of the total variance. Response options were scored on a 7‐point Likert scale ranging from 1 (strongly disagree)–7 (strongly agree). Higher scores indicate more positive attitudes. Items reflected the components of attitude, although more items addressed the cognitive domain (factor 1) than the affective and behavioural domains which merged into a second factor (factor 2) and six items were reverse‐scored. Social desirability may have influenced the results. However, most participants knew someone with ADRD, which was associated with more positive attitudes. These findings demonstrate the reliability and validity of the DAS, supporting its use as a research tool.

### 4.4 Analysis

The statistical analysis was carried out using SPSS V25.0. The following psychometric properties were evaluated: Structural validity was investigated by means of exploratory factor analysis (EFA) and Cronbach's alpha coefficient for inspecting the internal reliability testing (minimum acceptable value is 0.7). To explore structural validity, a principal component analysis (PCA) was used to extract factors to test whether the same factor structure was maintained as in the original English version. We took into account the following criteria to extract the factor components: the eigenvalues, which estimates the amount of variance subjected by each factor (minimum of 1), the scree plot (Cattell's test), which depicts different observable components, the Kaiser–Meyer–Olkin where measures sample adequacy (minimum acceptable value is 0.50) and the Bartlett's test of sphericity. Cronbach's alpha coefficient was measured separately for each determined factor. One‐way analysis of variance was also used to check differences between means of continuous variables for two different groups.

### 4.5 Ethics statements

This study was approved by the Ethical Committee of (Greek Association of Alzheimer's Disease and Related Disorders‐AADRD, Alzheimer Hellas). All procedures contributing to this work comply with the ethical standards outlined in the Declaration of Helsinki, which is relevant to the national and institutional committees on human experimentation. All students participated voluntarily in the study. They were informed about the procedure and the aim of the study and subsequently they provided their written consent for participation. Greek Law of Data Protection was respected through the confidentiality and anonymity of the data.

## 5 RESULTS

### 5.1 Developing the Greek version of questionnaires

In the current study, the translation–back translation method (Hambleton, 2001) was used to translate the English version of the DKAT, CODE and DAS into Greek. Specifically, two Greek experts translated separately the original English versions into Greek. Then, another two bilingual researchers in the UK performed the back translation from English to Greek. The originally translated and the back‐translated Greek versions were then compared for consistency, relevance and meaning of the content. Before the administration of the scale for the study demands, the pre‐final version was tested in 10 adults to detect any difficulties in understanding or any objections that they might have regarding the content. Because there were some objections regarding the understanding of the text, the problematic items were adapted by two authors (M.G and D.M.). After that, the scales were administered to five people to examine the adaptation of problematic items, finalizing the Greek version of the scales.

### 5.2 DAS‐GR

#### 5.2.1 Scale's validity

##### Factor structure

Structural validity, defined as the degree to which scores of a questionnaire are an adequate reflection of the dimensionality of the construct to be measured, was investigated by means of exploratory factor analysis (EFA). A principal component analysis (PCA) with Varimax rotation method was undertaken to extract factors for 210 participants. A minimum eigenvalue of 1 was specified as extraction criterion, and the criterion for factor loading was set at ≥0.30. Kaiser–Meyer–Olkin (KMO) measure of sampling adequacy was high with a value of 0.74, which indicates that the sampling is adequate. Bartlett's test of sphericity was significant, χ^2^(190) = 807.735, *p* < .001. This shows that the sample consists of related variables and EFA is there for suitable. The initial factor analysis revealed a six‐factor solution, which accounted for the 54.97% of the total variance. Eight items loaded the first factor, five items on the second factor, four items on third factor, four items on fourth factor, four items on fifth factor and four items on sixth factor. However, considering the Cattell's criterion (retain only those components above the point of inflection on a plot of eigenvalues ordered by diminishing size) only two factors were determined. The two factors were clearly identified and accounted for the 30.83% of the total variance. The first factor was labelled as “dementia comfort” (eigenvalue = 3.88), and the second factor was labelled as “knowledge about dementia” (eigenvalue = 2.28). Factor loading revealed that two items had loadings (≥0.30) on both factors (item 4: “I feel confident around people with ADRD,” “item 12: It is possible to enjoy interacting with people with ADRD”). We reran the analysis keeping item 12 in factor 2 and item 4 in factor 1 on the basis of their meaning and its closer relation to a specific factor. Table 1 displays the pattern coefficients for 20 items.

**Table 1:** Exploratory factor analysis of Dementia Attitudes Scale (DAS‐GR)

Items	Mean (*SD*)	Cronbach's α if item deleted	Factor 1 “Comfort”	Factor 2 “Knowledge”
13. I feel relaxed around people with ADRD.	4.37 (1.13)	0.673	0.702	
6. I feel uncomfortable being around people with ADRD*.	5.10 (1.30)	0.667	0.675	
2. I am afraid of people with ADRD*.	5.09 (1.51)	0.671	0.648	
17. I cannot imagine caring for someone with ADRD*.	4.90 (1.43)	0.682	0.586	
9. I would avoid an agitated person with ADRD*.	4.33 (1.51)	0.683	0.581	
4. I feel confident around people with ADRD.	4.61 (1.21)	0.698	0.511	
5. I am comfortable touching people with ADRD.	5.76 (1.40)	0.699	0.506	
8. I am not very familiar with ADRD*.	2.56 (1.80)	0.728	0.435	
16. I feel frustrated because I do not know how to help people with ADRD*.	3.18 (1.29)	0.728	0.318	
12. It is possible to enjoy interacting with people with ADRD.	0.4.27 (1.03)	0.646		0.410
18. I admire the coping skills of people with ADRD.	5.94 (0.98)	0.645		0.560
3. People with ADRD can be creative.	5.34 (1.24)	0.636		0.544
11. It is important to know the past history of people with ADRD.	6.37(0.81)	0.650		0.534
14. People with ADRD can enjoy life.	4.96 (1.30)	0.641		0.505
19. We can do a lot now to improve the lives of people with ADRD.	6.03 (0.92)	0.638		0.502
10. People with ADRD like having familiar things nearby.	5.77 (0.98)	0.644		0.472
20. Difficult behaviours may be a form of communication for people with ADRD.	4.88 (1.20)	0.648		0.449
15. People with ADRD can feel when others are kind to them.	5.61 (1.18)	0.656		0.389
7. Every person with ADRD has different needs.	6.36 (0.82)	0.667		0.348
1. It is rewarding to work with people who have ADRD.	5.77 (1.04)	0.662		0.336

* Reverse‐scored items.

Reliability of social comfort (factor 1) was α = 0.72 (good reliability) and α = 0.67 (relatively low reliability) for knowledge in dementia (factor 2). Thus, a two‐factor model seems most fitting. Furthermore, only few participants had a contact with person suffering from dementia (29,7%, *n* = 63). We investigated whether students with a previous contact with PwD had more positive attitudes. One‐way ANOVA demonstrated that there was a significant difference between students who were in contact with person suffering from dementia (*mean* = 104.11, *SD* 11.41) and those who were not (*mean* = 99.83, *SD* 9.22) in attitudes towards dementia scores, *F* (1,210) = 8.21, *p* = .005.

##### Scale's reliability

Internal consistency is the degree of the interrelatedness among items of the test. Cronbach's alpha coefficient for all 20 items of DAS was good (Cronbach's α = 0.74), suggesting very satisfactory internal consistency. That means that each item is correlated positively with the sum of the other items of the scale and significantly (all *p *< .0001).

### 5.3 DKAT2‐GR

#### 5.3.1 Scale's validity and reliability

Due to the specific structure of this tool, we did not use the factor analysis, but we explored the correct, incorrect and unsure answers, as participants were expected to have different levels of knowledge of dementia. A median of 9.5 questions were answered correctly (range 0–18) by 212 participants, and Cronbach's alpha coefficient was 0.68. There was no item that, if deleted, increased the value of Cronbach's alpha. Six items received a correct response from at least 65% of participants. Seven items received unsure response from at least 50% (Table 2).

**Table 2:** Percentages of correct and unsure responses to Dementia Knowledge Assessment Tool (DKAT2‐GR)

Correct statements	Participants % of correct responses	Participants % of unsure responses
1. Dementia because of changes in the brain.	78.8	18.9
2. Brain changes often progressive	83.9	15.2
3. Alzheimer's disease main cause.	17.5	52.4
4. Blood vessel disease can cause.	23.9	75.1
9. Limits life expectancy.	42.3	38.9
10. Families can help understand needs.	54.2	36.8
11. May develop problems with visual perception.	64.6	31.6
13. Uncharacteristic behaviours may occur.	70.3	28.8
14. Difficulty swallowing in late stage.	40.3	55.9
15. Movement limited in late stage.	65.1	30.2
17. May help to talk about feelings.	40.1	55.2
19. Can often be supported to make choices.	74.9	14.7
21. Exercise sometimes of benefit.	52.4	45.3

Incorrect statements*	Participants % of correct responses	Participants % of unsure responses
5. Confusion in older person almost always due to.	66.8	21.8
6. Only older adults develop dementia.	61.8	20.3
7. Knowing likely cause helps to predict.	10.0	44.1
8. Incontinence always in the early stages.	19.3	66.5
12. Sudden increases in confusion characteristic.	4.2	36.3
16. Changing environment makes no difference.	31.6	57.5
18. Important to always correct.	29.0	43.8
20. Impossible to tell if in pain.	22.2	66.0

* Reverse‐scored items.

#### 5.3.2 Item analysis

Two indices were calculated for each item: the difficulty index (percentage of correct answers) and the ignorance index (percentage of “I don't know” answers) (Waltz, Strickland, & Lenz, 2010). According to the difficulty index, the items were classified into six categories: very easy (>90%) correct answers; easy (75.1%–90%); somewhat easy (50.1%–75%); somewhat difficult (25.1%–50%); difficult (10.1%–25%); and very difficult (<10%). There were no items that were very easy, two items that were easy, eight items that were somewhat easy, five items that were somewhat difficult, five items that were difficult and one item that was very difficult.

### 5.4 CODE‐GR

#### 5.4.1 Scale's validity

Structural validity was investigated by means of exploratory factor analysis (EFA). A principal component analysis (PCA) with Varimax rotation method was applied to extract factor for 212 participants. A minimum eigenvalue of 1 was specified as extraction criterion, and the criterion for factor loading was set at ≥0.30. The overall KMO was 0.80, suggesting that the scale had good internal consistency without too much item redundancy. Bartlett's test of sphericity was significant with χ^2^(36) = 717.856, *p* < .001. The initial factor analysis revealed a two‐factor solution, which accounted for the 57.57% of the total variance. However, applying the Cattell's criterion, one factor was clearly identified and accounted for the 45,06% of the total variance. The factor considered as “knowledge in dementia” (eigenvalue = 4.05). Table 3 displays the pattern coefficients for the nine items.

**Table 3:** Exploratory factor analysis of Confidence in Dementia‐GR (CODE‐GR)

Items	Mean (*SD*)	Factor 1	Cronbach's α if item deleted
1. I feel able to understand the needs of a person with dementia when they cannot communicate well verbally	2.18 (1.05)	0.641	0.832
2. I feel able to interact with a person with dementia when they cannot communicate well verbally	2.40 (1.00)	0.756	0.819
3. I feel able to manage situations when a person with dementia becomes agitated	1.62 (0.92)	0.641	0.841
4. I feel able to identify when a person may have a dementia	2.50 (0.95)	0.450	0.818
5. I feel able to gather relevant information to understand the needs of a person with dementia	2.81 (1.07)	0.682	0.825
6. I feel able to help a person with dementia feel safe during their stay in hospital	2.75 (1.11)	0.711	0.827
7. I feel able to work with people who have a diagnosis of dementia	1.88 (1.05)	0.681	0.826
8. I feel able to understand the needs of a person with dementia when they can communicate well verbally	3.20 (1.07)	0.712	0.824
9. I feel able to interact with a person with dementia when they can communicate well verbally	3.44 (1.00)	0.720	0.823

### 5.5 Scale's reliability

#### 5.5.1 Internal consistency

Cronbach's alpha coefficient for all nine items of CODE‐GR was high (Cronbach's α = 0.85), suggesting very satisfactory internal consistency. That means that each item is correlated positively with the sum of the other items of the scale and significantly (all *ps* < 0.0001).

### 5.6 Correlations among questionnaires

Convergent validity of questionnaires was testing. CODE‐GR had a medium positive correlation (*r* = .363**, *p* < .001) with DAS‐GR (comfort) demonstrating that confidence is positively correlated with attitudes. Additionally, DAS‐GR (knowledge) had a negative correlation with two questions of DKAT2‐GR scale (item 17: “May help to talk about feelings,” *r* = −.253**, *p* < .001, item 21: “Exercise sometimes of benefit,” *r* = −.202, *p* < .001). The negative correlation here is explained due to coding of variables. By these results, we can assume that positive attitudes are related to higher knowledge about dementia and confidence in provision of care people with dementia. However, no other significant relationship was observed between CODE‐GR and DKAT2‐GR, demonstrating that there is no direct relation between knowledge and confidence in caring people with dementia.

## 6 DISCUSSION

In the current cross‐sectional study, DAS, DKAT and CODE Scale were validated on the Greek population, by means of exploring their psychometric properties. Our goal was to use these scales for evaluating a future dementia programme in general hospitals. The Greek versions were found to have high internal reliability for CODE‐GR, adequate reliability for DAS‐GR and acceptable reliability for DKAT2‐GR. Construct validity was satisfactory for DAS‐GR, DKAT2‐GR and CODE‐GR. Their convergent validity was partially supported to this study. DAS‐GR (knowledge) was highly correlated with two items of the DKAT2‐GR scale, supporting the relationship between knowledge and attitudes towards dementia as demonstrated also in the literature (Evripidou, Charalambous, Middleton, & Papastavrou, 2019). Besides, DAS‐GR (comfort) was highly correlated with the CODE‐GR scale. Thus, self‐confidence in caring PwD significantly correlates with more positive attitudes towards people with dementia (Travers, Beattie, Martin‐Khan, & Fielding, 2013). It has been mentioned that DAS and DKAT2 had reverse‐scored items and this may has contributed to a negative impact on the psychometric properties of scales. Τhere is an increasing amount of literature that reports problems with reverse‐scored items (Lindwall et al., 2012). However, the extent to which reverse‐scored items control for response sets is debatable (Comrey, 1988).

The psychometric properties of the DAS‐GR are compared favourably with the psychometric properties of similar scales (Lintern & Woods, 2000; Thomas, Palmer, Coker‐Juneau, & Williams, 2003), and it appears to be a useful tool for assessing attitudes towards dementia not only in students (Lokon, Li, & Parajuli, 2017; Kimzey et al., 2016) but also in staff health care (Scerri & Scerri, 2017). The DAS‐GR has an acceptable Cronbach (0.74), but lower than the original version (a = 0.83–0.85) (both studies used students’ sample). Concerning factor analysis, the DAS‐GR scale identified two factors, social comfort and knowledge, likely with its original version (O’Connor & McFadden, 2010) but some items loaded in different factors from the original study. Αpart from the validation done by O’Connor and McFadden (2010) in the United States, the DAS has so far been translated and validated in Croatia (care workers and general population samples) (Ćoso & Mavrinac, 2016) and Netherlands (general population sample) (Veer, 2018) demonstrating good psychometric properties. In specific, factor analysis in Croatian and Dutch versions revealed almost identical structure as in the original study and current study with two factors solution (social comfort and dementia knowledge). Internal consistency for Croatian DAS (α = 0.85) and Dutch DAS (“comfort in dementia,” α = 0.82 and “knowledge in dementia,” α = 0.75) was higher compared to the Greek version. Besides, the Croatian validation of the DAS identified a positive–negative structure in a sample of employees and professionals that have everyday contact with people with dementia. A similar positive–negative structure was found among the sample of the general population of the Dutch version. The current study also found a positive association between previous contact of students with PwD and attitudes which is in accordance to literature (Ahmad Basri, Subramaniam, Ghazali, & Ajit Singh, 2017). Conclusively, the DAS is a useful tool used in research (Kimzey et al., 2016; Roberts & Noble, 2015; Scerri & Scerri, 2013) and its strengths include reliability, convergent validity evidence, practical length and ease of administration. Moreover, there are few questionnaires that deal with attitudes towards dementia (like AQD), but the DAS is a more general tool and the only one that covers the entire construct of dementia (Ćoso & Mavrinac, 2016).

The DKAT2‐GR showed good psychometric properties as regards validity and reliability, being useful to evaluate knowledge of dementia in those providing care. Internal consistency was almost acceptable (α = 0.68) but lower than that found in the original study (α = 0.79 for both family and staff carers) (Toye et al., 2013). Our results fit well with those found in the original publication (Toye et al., 2013) and with the Spanish (Parra‐Anguita, Moreno‐Cámara, López‐Franco, & Pancorbo‐Hidalgo, 2018) validation. In Spanish version, internal consistency was higher compared to the current study (Cronbach's alpha = 0.76 for nursing professionals and 0.83 for students) (Parra‐Anguita et al., 2018). However, items’ analysis in Spanish student's sample revealed similar indices of difficulty compared to the current study. Particularly, there were no very easy items and the most of them were somewhat easy or somewhat difficult. Nevertheless, more Greek students found some items difficult, compared to Spanish sample of students.

The psychometric properties of the DKAT2‐GR are compared favourably with the psychometric properties of similar scales (ADKT (Dieckmann et al., 1988), UAB‐ADKT (Barrett JJ et al., 1997), DQ (Gilleard & Groom, 1994), KAML‐C (Kuhn et al., 2005) ADKS (Carpenter, Balsis, Otilingam, Hanson, & Gatz, 2009) and DK‐20 (Shanahan, Orrell, Schepers, & Spector, 2013)), indicating an acceptable reliability. Moreover, DKAT2 is positively correlated (*r*'s = .32–.45) with other knowledge measures used in staff care (ADKT, ADKS, DK‐ 20) demonstrating convergent validity (Sullivan & Mullan, 2017). Comparing DKAT2 with other measures, it must be mentioned that ADKT is the most widely used tool in research context about knowledge. Although the psychometric properties of ADKT have established and once suited to international use, some items are now outdated (Spector et al., 2012). Although ADKS is an updated version of the ADKT and is also a widely used tool (Kimzey et al., 2016; Scerri & Scerri, 2013; Eshbaugh, 2014; Kada, 2015), both scales measure knowledge towards Alzheimer disease lacking a broader use in other dementia‐related disorders. The UAB‐ADKT and the DQ are less used in research, while the DQ is most suitable for family carers (Spector et al., 2012). Thus, DKAT2 appears to be a useful tool in research (Eccleston et al., 2015; Robinson et al., 2014) for assessing knowledge towards dementia in professional staff and has the ability to differentiate correctly between people with high or low knowledge.

CODE‐GR demonstrated very satisfactory psychometric properties being useful tool to evaluate confidence in dementia professional care. The current findings support the results from the initial study (nurse staff sample). In particular, internal consistency was very good (α = 0.85), slightly lower than that found in the original study (α = 0.91) and that found in another study conducted by its developers, where a greater number of nurse staff were included, combining data from the original study (α = 0.88) (Elvish et al., 2018). In the current study, CODE is a scale comprising of one factor which is in accordance to its developers’ validation (Elvish et al., 2014; Elvish et al., 2018). Although there are a limited number of questionnaires about confidence in dementia care, CODE Scale is adequately used in research (Elvish et al., 2014; Elvish et al., 2018; Scerri & Scerri, 2017) and fits favourably with the psychometric properties of other confidence scales (Palmer et al., 2014) or other similar scales of self‐efficacy (Mackenzie & Peragine, 2003; Schindel Martin & Dupuis, 2005; Coates, 1997).

As population ages and dementia increases, the hospital care for PwD will continue to be considerable. Effective training programmes appeared to be longer than 8 hr’ duration and were classroom‐based. Training content must be relevant to participants’ work, while practical tools and care strategies must be directly applicable in their workplace (Surr et al., 2017). Moreover, the content should focus on behavioural management like communication and personal‐centred techniques, leading to better understanding of patients’ needs and to increase the confidence level to individualized dementia care (Evripidou et al., 2019).

The need for staff training at workplaces is significant but may be more significant to take place at an early stage of nursing education, so as for nurses to apply this advanced knowledge to their clinical routine of dementia care (Evripidou et al., 2019). However, staff adequacy, nurse leadership, work environment, education, age and level of communication skills (Elvish et al., 2016; Shinan‐Altman, Werner, & Cohen 2014; Kada, Nygaard, Mukesh, & Geitung, 2009; Fessey, 2007) are some additional factors that influence attitudes as training outcomes. Training programmes supposed to provide a higher level of knowledge in staff members, which is correlated with higher confidence level and positive attitudes towards people with dementia (Evripidou et al., 2019). The DAS, CODE Scale and DKAT were translated and validated in Greek for this purpose.

## 7 LIMITATIONS AND FUTURE RESEARCH

This study has some limitations as DAS, DKAT and CODE are self‐report questionnaires. Besides, our sample was comprised of students, primarily young adults, so generalization of the results is limited. Additional research should include a target group of healthcare staff where a test–retest design is strongly recommended. DAS‐GR, DKAT2‐GR and CODE‐GR should include a confirmatory factor analysis with larger samples. Further convergent validity testing should be conducted between the DAS‐GR, DKAT2‐GR and CODE‐GR and other similar scales measuring attitudes towards dementia (such as the ADQ), knowledge in dementia (such as ADKT) and confidence in providing care (such as SBMSEP), but this was beyond the scope of the current research. Future evidence is needed for the replicability of the factor structure across independent samples in relation to DAS measure.

## 8 CONCLUSIONS

- Evidence for the psychometric properties of the DAS‐GR, CODE‐GR and DKAT2‐GR supports their use as valid and robust measurements in Greek research context.
- All three scales can be used as research tools, evaluating future dementia trainings in healthcare settings.
- Results from larger studies or studies with different populations can be further contributed in this field.
- Use of three scales creates a possibility to compare further study results with other studies conducted in other languages.

## FUNDING

This work was supported by the Robert Bosch Foundation. This is independent research. The views expressed in this publication are those of the authors.

## Supporting information

Supplementary File
