# Supplementary material for: Psychometric properties of dementia attitudes scale, dementia knowledge assessment tool 2 and confidence in dementia scale in a Greek sample
Source: Nurs Open. 2020 Jul 3;7(5):1623–33. doi: 10.1002/nop2.546 (PMC7424436; doi:10.1002/nop2.546)
Supplement: Supplementary file 1 — Supplementary File [file NOP2-7-1623-s001.docx]

Supplement File 1.

Strengthening the Reporting of observational Studies in Epidemiology (STROBE) checklist

|  | **Item No** | **Reccomendation** | **Page** |
| --- | --- | --- | --- |
| **Title and abstract** | 1 | (*a*) Indicate the study’s design with a commonly used term in the title or the abstract | 1 |
|  |  | (*b*) Provide in the abstract an informative and balanced summary of what was done  and what was found | 1 |
| **Introduction** |  |  |  |
| Background/rationale | 2 | Explain the scientific background and rationale for the investigation being reported | 1-3 |
| Objectives | 3 | State specific objectives, including any prespecified hypotheses | 3 |
| **Methods** |  |  |  |
| Study design | 4 | Present key elements of study design early in the paper | 3 |
| Setting | 5 | Describe the setting, locations, and relevant dates, including periods of recruitment,  exposure, follow-up, and data collection | 3 |
| Participants | 6 | (*a*) *Cohort study*—Give the eligibility criteria, and the sources and methods of  selection of participants. Describe methods of follow-up  *Case-control study*—Give the eligibility criteria, and the sources and methods of  case ascertainment and control selection. Give the rationale for the choice of cases  and controls  *Cross-sectional study*—Give the eligibility criteria, and the sources and methods of  selection of participants  (*b*) *Cohort study*—For matched studies, give matching criteria and number of  exposed and unexposed  *Case-control study*—For matched studies, give matching criteria and the number of  controls per case | 3 |
| Variables | 7 | Clearly define all outcomes, exposures, predictors, potential confounders, and effect  modifiers. Give diagnostic criteria, if applicable | 3-4 |
| Data sources/measurement | 8 | For each variable of interest, give sources of data and details of methods of  assessment (measurement). Describe comparability of assessment methods if there  is more than one group | 3-4 |
| Bias | 9 | Describe any efforts to address potential sources of bias | 6-7 |
| Study size | 10 | Explain how the study size was arrived at | 3 |
| Quantitative variables | 11 | Explain how quantitative variables were handled in the analyses. If applicable, describe which groupings were chosen and why | 3,4,6 |
| Statistical methods | 12 | (*a*) Describe all statistical methods, including those used to control for confounding  (*b*) Describe any methods used to examine subgroups and interactions  (*c*) Explain how missing data were addressed  (*d*) *Cohort study*—If applicable, explain how loss to follow-up was addressed  *Case-control study*—If applicable, explain how matching of cases and controls was  addressed  *Cross-sectional study*—If applicable, describe analytical methods taking account of  sampling strategy  (*e*) Describe any sensitivity analyses | 4 |
| **Results** |  |  |  |
|  | 13 | (a) Report numbers of individuals at each stage of study—eg numbers potentially eligible, examined for eligibility, confirmed eligible, included in the study, completing follow-up, and analysed  (b) Give reasons for non-participation at each stage  (c) Consider use of a flow diagram | 4-6 |
| Descriptive  data | 14 | (a) Give characteristics of study participants (eg demographic, clinical, social) and information  on exposures and potential confounders  (b) Indicate number of participants with missing data for each variable of interest  (c) *Cohort study*—Summarise follow-up time (eg, average and total amount) | a 3  b 5-6 |
| Outcome data | 15 | *Cohort study*—Report numbers of outcome events or summary measures over time  *Case-control study—*Report numbers in each exposure category, or summary measures of  exposure  *Cross-sectional study—*Report numbers of outcome events or summary measures | 5-6 |
| Main results | 16 | (*a*) Give unadjusted estimates and, if applicable, confounder-adjusted estimates and their  precision (eg, 95% confidence interval). Make clear which confounders were adjusted for and  why they were included  (*b*) Report category boundaries when continuous variables were categorized  (*c*) If relevant, consider translating estimates of relative risk into absolute risk for a meaningful  time period | 5-6 |
| Other analysis | 17 | Report other analyses done—eg analyses of subgroups and interactions, and sensitivity  analyses | - |
| **Discussion** |  |  |  |
| Key results | 18 | Summarise key results with reference to study objectives | 6-7 |
| Limitation | 19 | Discuss limitations of the study, taking into account sources of potential bias or imprecision.  Discuss both direction and magnitude of any potential bias | 9 |
| Interpratation | 20 | Give a cautious overall interpretation of results considering objectives, limitations, multiplicity  of analyses, results from similar studies, and other relevant evidence | 6-9 |
| Generalisability | 21 | Discuss the generalisability (external validity) of the study results | 9 |
| **Other information** |  |  |  |
| Funding | 22 | Give the source of funding and the role of the funders for the present study and, if applicable,  for the original study on which the present article is based | 9 |

*Give information separately for cases and controls in case-control studies and, if applicable, for exposed and

unexposed groups in cohort and cross-sectional studies.
